# Lipid biosynthesis perturbation impairs endoplasmic reticulum–associated degradation

**DOI:** 10.1016/j.jbc.2023.104939

**Published:** 2023-06-17

**Authors:** Samantha M. Turk, Christopher J. Indovina, Jacob M. Miller, Danielle L. Overton, Avery M. Runnebohm, Cade J. Orchard, Mary E. Tragesser-Tiña, Samantha K. Gosser, Ellen M. Doss, Kyle A. Richards, Courtney Broshar Irelan, Mahmoud M. Daraghmi, Connor G. Bailey, Julia M. Niekamp, Kieran P. Claypool, Sarah M. Engle, Bryce W. Buchanan, Kelsey A. Woodruff, James B. Olesen, Philip J. Smaldino, Eric M. Rubenstein

**Affiliations:** Department of Biology, Ball State University, Muncie, Indiana, USA

**Keywords:** ER quality control, translocon quality control, endoplasmic reticulum-associated degradation (ERAD), protein degradation, phospholipid metabolism, sterol, *Saccharomyces cerevisiae*, yeast genetics, Hrd1, Doa10

## Abstract

The relationship between lipid homeostasis and protein homeostasis (proteostasis) is complex and remains incompletely understood. We conducted a screen for genes required for efficient degradation of *Deg1*-Sec62, a model aberrant translocon-associated substrate of the endoplasmic reticulum (ER) ubiquitin ligase Hrd1, in *Saccharomyces cerevisiae*. This screen revealed that *INO4* is required for efficient *Deg1*-Sec62 degradation. *INO4* encodes one subunit of the Ino2/Ino4 heterodimeric transcription factor, which regulates expression of genes required for lipid biosynthesis. *Deg1*-Sec62 degradation was also impaired by mutation of genes encoding several enzymes mediating phospholipid and sterol biosynthesis. The degradation defect in *ino4*Δ yeast was rescued by supplementation with metabolites whose synthesis and uptake are mediated by Ino2/Ino4 targets. Stabilization of a panel of substrates of the Hrd1 and Doa10 ER ubiquitin ligases by *INO4* deletion indicates ER protein quality control is generally sensitive to perturbed lipid homeostasis. Loss of *INO4* sensitized yeast to proteotoxic stress, suggesting a broad requirement for lipid homeostasis in maintaining proteostasis. A better understanding of the dynamic relationship between lipid homeostasis and proteostasis may lead to improved understanding and treatment of several human diseases associated with altered lipid biosynthesis.

Proteome maintenance is crucial for eukaryotic life. Dedicated mechanisms to destroy aberrant or overabundant proteins are present in many cellular compartments. A substantial proportion of protein turnover at the endoplasmic reticulum (ER) is accomplished through ER-Associated Degradation (ERAD; reviewed in (1, 2)). In ERAD, ubiquitin ligases transfer ubiquitin from ubiquitin-conjugating enzymes to aberrant or overabundant proteins, which are subsequently degraded by the 26S proteasome. Mechanisms of ERAD are highly conserved among eukaryotes, and many genetic and mechanistic advances in understanding this system were first made in *Saccharomyces cerevisiae* (3). The two major ERAD ubiquitin ligases in *S. cerevisiae* are the highly conserved multipass transmembrane enzymes, Hrd1 and Doa10 (4, 5, 6). Hrd1 functions with the soluble ubiquitin-conjugating enzyme Ubc7 and, to a lesser extent, Ubc1 and Ubc6 (5, 7, 8). Doa10 functions with two ubiquitin-conjugating enzymes, Ubc7 and the transmembrane protein Ubc6 (6). Ubc7 is tethered to the ER membrane and stabilized by the transmembrane protein Cue1 (9, 10, 11).

Hrd1 and Doa10 differentially target ERAD substrates based on the location and nature of the substrates’ degrons, or degradation signals. In addition to targeting soluble and integral membrane proteins for degradation, Hrd1 promotes translocon quality control (TQC), whereby the enzyme ubiquitylates proteins that clog translocons, channels that transfer proteins into or across the ER membrane (12, 13, 14, 15). Conversely, Doa10 recognizes soluble and integral membrane proteins with cytosolic degrons (16, 17, 18). Both enzymes target proteins with intramembrane degrons (19, 20). While Hrd1 resides exclusively in the ER membrane, Doa10 is also found in the contiguous inner nuclear membrane (INM), where it ubiquitylates proteins with nucleoplasmic degrons (21). Additional ubiquitin ligases contribute to the degradation of proteins at the ER and INM. The ubiquitin ligases Ubr1 and Ltn1 contribute to ERAD (22, 23, 24), and the INM-Asi complex and anaphase-promoting complex mediate the turnover of aberrant or overabundant INM proteins (25, 26, 27). Finally, the metalloprotease Ste24 contributes to TQC via a mechanism that is partially redundant with Hrd1-mediated ubiquitylation (15, 28).

Molecular mechanisms of ERAD of proteins with luminal, transmembrane, and cytosolic degrons have been extensively characterized in yeast and mammals. By contrast, comprehensive characterization of genetic requirements for the degradation of proteins that persistently engage translocons remains incomplete. We conducted a genome-wide, growth-based reporter screen to identify yeast genes required for the turnover of a model translocon-associated substrate of Hrd1. This screen revealed that *INO4* is required for efficient TQC substrate degradation. *INO4* encodes one subunit of a heterodimeric transcription factor that regulates several genes encoding lipid-biosynthetic enzymes (29, 30). We found TQC is broadly sensitive to perturbations in phospholipid and sterol biosynthesis. Further, a panel of model Hrd1 and Doa10 substrates bearing luminal, intramembrane, and cytosolic degrons were stabilized by *INO4* deletion, and yeast with defects in phospholipid or sterol synthesis were sensitive to conditions associated with aberrant protein production. The abundance of Ubc7, which is broadly required for ERAD, was reduced in *ino4*Δ yeast, suggesting a possible mechanism for disrupted ERAD. Taken together, our results indicate that altered lipid homeostasis broadly and profoundly impairs ER proteostasis. Several metabolic, muscular, cardiac, and neurodegenerative diseases are associated with perturbed lipid synthesis (31, 32, 33, 34, 35, 36). Altered lipid homeostasis may impair ER protein degradation in individuals with these disorders.

## Results

### Screen to identify genes required for degradation of model translocon quality control substrate

**Figure 1:**
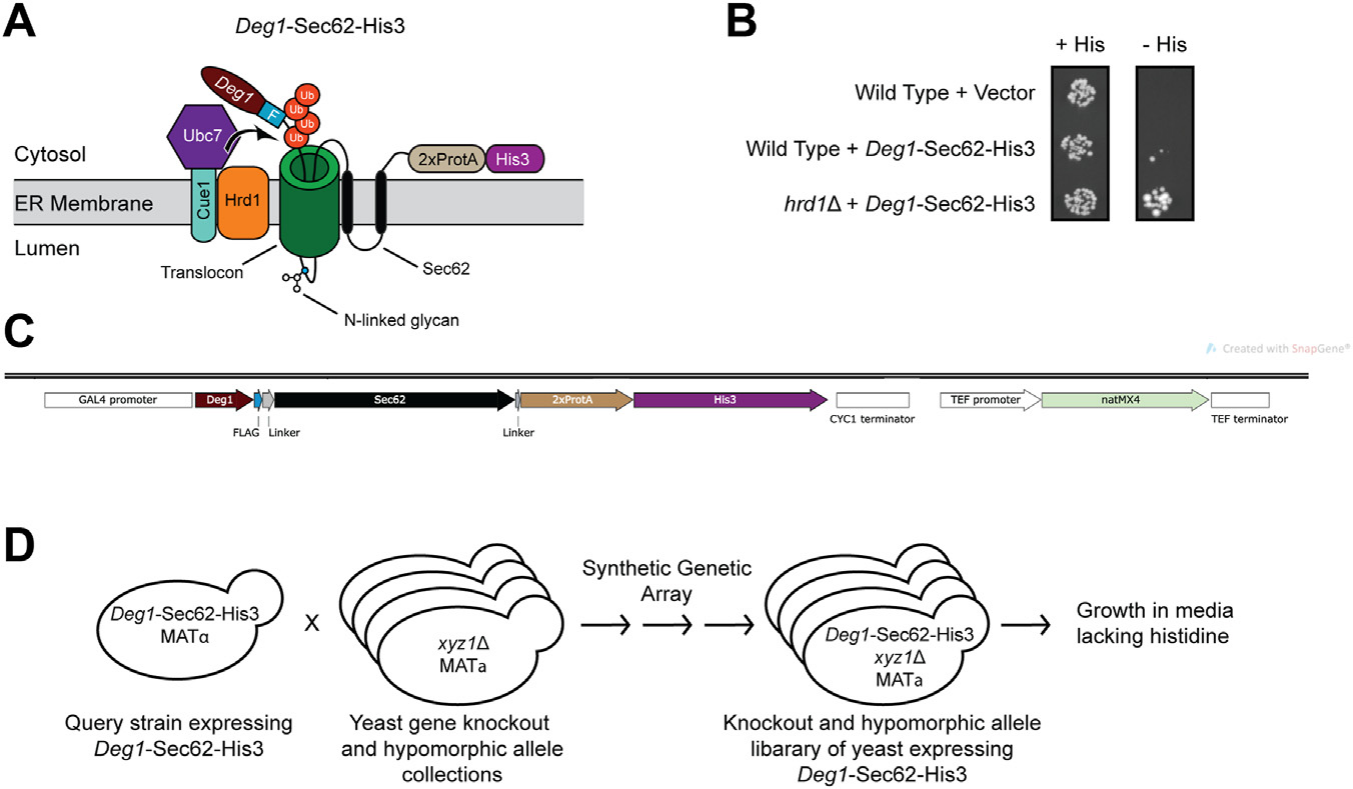
**Screen for genes required for degradation of a model translocon-associated protein.***A*, schematic of *Deg1*-Sec62-His3 following aberrant translocon engagement. Following the integration of the two transmembrane segments of Sec62, the N-terminal tail of the fusion protein loops into and persistently engages (*i.e.*, clogs) the translocon (14). Upon clogging, *Deg1*-Sec62(±His3) undergoes N-linked glycosylation and is ubiquitylated by Hrd1 and Ubc7 (which is anchored at the ER membrane by Cue1). *Deg1*-Sec62-His3 possesses, in sequence, *Deg1* (the N-terminal 67 amino acids from the yeast transcriptional repressor MATα2), a FLAG epitope (F), Sec62, two copies of *Staphylococcus aureus* Protein A (2xProtA), and the His3 enzyme. Ub, ubiquitin. *B*, yeast of the indicated genotypes transformed with an empty vector or a plasmid encoding *Deg1*-Sec62-His3 were spotted onto media containing or lacking histidine (His). *C*, *DOA10* locus of the query strain used for the genome-wide screen. *DOA10* was replaced with a cassette containing *Deg1-Sec62-His3* and *natMX4* as two independent genes, each with its own promoter and transcriptional terminator. *D*, overview of the genome-wide screen. See text and Table 1 for details.

We conducted a reporter-based screen to identify genes required for efficient degradation of the model TQC substrate *Deg1*-Sec62. Fusing His3 to the C-terminus of *Deg1*-Sec62 (Fig. 1*A*) allows the selection of degradation-defective mutant yeast lacking endogenous *HIS3*. Yeast unable to degrade *Deg1*-Sec62-His3 exhibits histidine prototrophy ((37), Fig. 1*B*).

A query strain encoding *Deg1*-Sec62-His3 driven by the *GAL4* promoter (Fig. 1*C*) was crossed with collections of yeast strains possessing deletions of non-essential genes and hypomorphic alleles of essential genes. Using Synthetic Genetic Array (SGA) technology (38), a library of ∼6000 unique mutant strains harboring *Deg1*-Sec62-His3 was generated (Fig. 1*D* and Table 1). Under some conditions (*e.g.*, when ER translocation is impaired), *Deg1*-Sec62 is converted from a Hrd1 substrate into a Doa10 substrate (14). Therefore, to simplify the analysis, the gene encoding *Deg1*-Sec62-His3 was introduced at the *DOA10* locus, replacing *DOA10* in the query strain.

**Table 1:** Detailed outline of Synthetic Genetic Array (SGA) screen procedure

Description	Media	Agar or liquid media	Time
Prepare a lawn of query strain (VJY355)^a^	YPD	Agar	2 days
Pin array of library strains from 96-well plate of cryopreserved yeast	YPD	Agar	2 days
Mate yeast by replica-pinning query strain and library strains onto fresh plate	YPD	Agar	1 day
Mated diploid selection^b^	YPD + 100 μg/ml nourseothricin (clonNAT; WERNER BioAgents) + 200 μg/ml G418 (G418 Sulfate; CALBIOCHEM)	Agar	2 days
Sporulation^c^	1% Potassium Acetate, 0.1% yeast extract, 0.05% glucose, 0.002% adenine, 0.004% uracil, 0.0005% arginine, 0.00025% histidine, 0.0015% isoleucine, 0.0015% leucine, 0.001% lysine, 0.00025% methionine, 0.0015% phenylalanine, 0.00125% threonine, 0.001% tryptophan	Agar	5–7 days
Haploid MATa selection^d^	0.67% yeast extract without amino acids, 2% glucose, 0.0005% arginine, 0.00025% histidine, 0.0015% isoleucine, 0.0015% leucine, 0.001% lysine, 0.00025% methionine, 0.0015% phenylalanine, 0.00125% threonine, 0.001% tryptophan, 0.004% adenine, 0.008% uracil, 50 μg/ml canavanine; 50 μg/ml thialysine	Agar	2 days
Gene deletion or hypomorphic allele selection^e^	MATa selection media + 200 μg/ml G418	Agar	2 days
Selection of deletion or hypomorphic allele *AND Deg1-Sec62-His3:natMX4* reporter^f^	MATa selection media + 200 μg/ml G418 + 100 μg/ml nourseothricin	Agar	2 days
Prepare 96-well plates with liquid cultures	MATa selection media + 200 μg/ml G418 + 100 μg/ml nourseothricin	Liquid	2 days
Transfer 40 μl of liquid culture to fresh media to select for genes required for *Deg1*-Sec62-His3 degradation	MATa selection media + 200 μg/ml G418 + 100 μg/ml nourseothricin *without* histidine	Liquid	11 h (Record OD_595_ at beginning and end of 11 h)

a All steps were performed at 30 °C, except for mating and sporulation, which were performed at 23 °C. All transfers (except final transfer to screen media) were performed using sterile 96-pronged pinners.

b The query strain locus encoding *Deg1*-Sec62-His3 also contains the *natMX4* nourseothricin-resistance gene. All deletion and hypomorphic library strains possess the *kanMX4* gene, which confers resistance to G418. Only mated diploid strains may grow in the presence of both nourseothricin and G418.

c Sporulation was induced by culturing yeast on media with limited nitrogen and carbon (106).

d Haploid selection was mediated by toxic amino acid analogs thialysine and canavanine, which enter yeast *viaLYP1* and *CAN1* gene products, respectively. The query strain possesses *LYP1* and *CAN1* deletions, while screened library strains possess wild type alleles of these genes. Heterozygous *LYP1*/*lyp1*Δ *CAN1*/*can1*Δ mated diploid yeast are susceptible to thialysine and canavanine. Only haploid *lyp1*Δ *can1*Δ yeast can survive on such media. Furthermore, in the query strain, the *CAN1* gene was replaced with the *LEU2* gene driven by the promoter for the MATa-specific gene, *STE2*, which allowed only MATa cells to produce leucine and survive in the absence of exogenously provided leucine.

e Continued presence of G418 ensures preservation and selection of yeast with library deletion or hypomorphic alleles.

f Continued presence of nourseothricin ensures preservation and selection of yeast possessing *Deg1*-Sec62-His3:*natMX4*.

Each mutant strain with *Deg1*-Sec62-His3 was inoculated into liquid media (containing histidine) in a 96-well plate and allowed to incubate at 30 °C for 48 h. Equal volumes of each culture were transferred to fresh media lacking histidine and incubated for 11 h at 30 °C. The optical density at 595 nm (OD_595_) of each strain was recorded at the beginning and end of the 11 h incubation period. A cutoff for ΔOD_595_ values of 0.079 was selected, resulting in 128 genes encoding proteins with potential roles in *Deg1*-Sec62-His3 degradation (File S1). Deletion of *GAL80* (39), which encodes a repressor of the *GAL4* promoter used to drive expression of *Deg1*-Sec62-His3, yielded the highest ΔOD_595_ value. *HRD1*, *HRD3* (which encodes a Hrd1 cofactor (4, 14)), and *UMP1* (which encodes a proteasome assembly factor (40)) were identified in this screen, providing confidence in the power of this analysis to yield bona fide genetic requirements for protein degradation.

Gene Ontology (GO) analysis (www.yeastgenome.org) of the 128 genes revealed significant enrichment of genes linked to processes related to sulfur metabolism (*sulfate assimilation*, *sulfur compound biosynthetic process*, *sulfur amino acid metabolic process*, *hydrogen sulfide metabolic process*, and *hydrogen sulfide biosynthetic process*) (Table 2). No GO terms relating to function or component were significantly enriched.

**Table 2:** Gene Ontology (GO) Process Term analysis for genes identified in genome-wide screen

Gene Ontology Term	Cluster frequency	Genome frequency	Corrected *p* value	Genes
sulfate assimilation	5 of 128 genes, 3.9%	9 of 7166 genes, 0.1%	0.00012	*MET3 MET5 MET8 MET10 MET14*
sulfur compound biosynthetic process	10 of 128 genes, 7.8%	82 of 7166 genes, 1.1%	0.00102	*HOM2 HOM3 MET2 MET3 MET5 MET6 MET10 MET14 PDA1 SAM1*
sulfur amino acid metabolic process	8 of 128 genes, 6.2%	49 of 7166 genes, 0.7%	0.00124	*HOM2 HOM3 MET2 MET3 MET5 MET6 MET14 SAM1*
hydrogen sulfide metabolic process	4 of 128 genes, 3.1%	8 of 7166 genes, 0.1%	0.00395	*MET3 MET5 MET10 MET14*
hydrogen sulfide biosynthetic process	4 of 128 genes, 3.1%	8 of 7166 genes, 0.1%	0.00395	*MET3 MET5 MET10 MET14*

128 genes with ΔOD_595_ values greater than or equal to 0.079 in screen for genes with potential roles in ER protein degradation were analyzed using the Gene Ontology Term Finder at the *Saccharomyces* Genome Database (https://www.yeastgenome.org/goTermFinder) using a *p*-value cutoff of 0.01. No Function or Component GO Terms were significantly enriched for the input list of 128 genes.

A majority (88 of 128) of mutants yielding ΔOD_595_ values at or above the 0.079 cutoff were selected for further evaluation. Naive yeast with mutations in genes identified in the screen was transformed with plasmids encoding *Deg1*-Sec62-His3 and/or *Deg1*∗-Sec62 for confirmatory reporter-based growth assays and/or biochemical analysis (*i.e.*, cycloheximide chase experiments and western blots), respectively, as indicated in File S1. *Deg1*∗-Sec62 possesses mutations that preclude degradation by the Doa10 pathway while still permitting Hrd1-mediated degradation (14, 41). Reasons for excluding specific mutants from further analysis are outlined in File S1 (*e.g.*, genes encoding products with non-specific roles in gene expression were not assayed).

**Figure 2:**
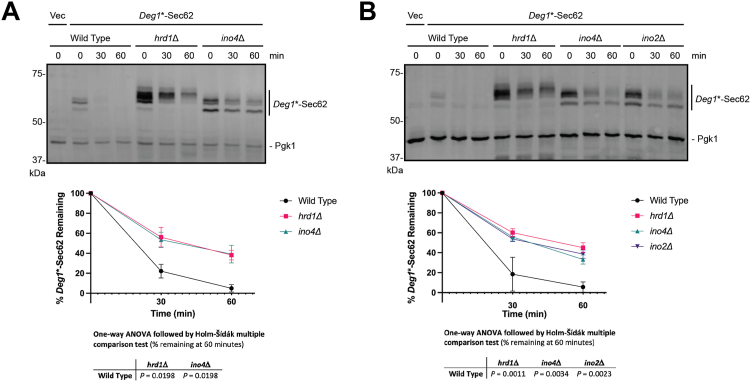
**Ino2 and Ino4 are required for Deg1∗-Sec62 degradation.***A*, WT yeast or yeast lacking either HRD1 or INO4 were transformed with a plasmid encoding Deg1∗-Sec62 or an empty vector and subjected to cycloheximide chase and western blot analysis to detect Deg1∗-Sec62 and Pgk1. *B*, as in (*A*), but with WT yeast or yeast lacking either HRD1, INO4, or INO2. Means of percent Deg1∗-Sec62 remaining for three to four biological replicates are plotted. Error bars represent the SEM. Means of percent Deg1∗-Sec62 remaining at 60 min were evaluated by one-way ANOVA followed by Holm-Šídák multiple comparison tests (only pairs relative to WT yeast were compared).

Thirty of seventy five mutants retested by the reporter-based growth assay recapitulated the screen results (*i.e.*, enhanced growth in the absence of histidine). Of 41 gene mutations tested by cycloheximide chase and western blot (which included mutations that were confirmed by growth assay and mutations that were selected directly for biochemical analysis), loss of four enhanced stability of *Deg1*∗-Sec62 (*HRD1*, *INO4*, *KAR3*, and *SET2*). Mutation of 15 genes increased steady state *Deg1*∗-Sec62 abundance without detectably impacting degradation kinetics (abundance fold increase in mutants relative to wild type yeast is presented in File S1). Deletion of *INO4* and *KAR3* (which encodes a minus-end-directed kinesin) strongly stabilized *Deg1*∗-Sec62, while deletion of *SET2* (which encodes a histone methyltransferase) modestly, but reproducibly, delayed *Deg1*∗-Sec62 turnover (Fig. 2*A* and Fig. S1, *A* and *B*). An example of a gene whose mutation increased the steady state abundance of *Deg1*∗-Sec62 without delaying degradation is *YDJ1* (Fig. S1*C*). *INO4*, *KAR3*, and *SET2* have not previously been implicated in TQC.

### *INO2* and *INO4* are required for efficient degradation of *Deg1*∗-Sec62

The Ino2/Ino4 heterodimeric transcription factor regulates the expression of at least 88 genes, many of which encode enzymes involved in phospholipid synthesis (29, 30). To our knowledge, the Ino2/Ino4 complex has not previously been implicated in ERAD. Cycloheximide chase analysis confirmed that loss of *INO4* stabilizes *Deg1*∗-Sec62 to a similar extent as *HRD1* deletion (Fig. 2*A*). *Deg1*∗-Sec62 migrates as multiple bands; appearance of higher molecular weight species reflects N-linked glycosylation of the protein, which occurs upon persistent translocon engagement (14). In addition to slowing degradation of *Deg1*∗-Sec62, *INO4* deletion delays the appearance of higher molecular weight species. Loss of Ino4’s binding partner Ino2 similarly stabilized and delayed modification of *Deg1*∗-Sec62 (Fig. 2*B*).

### Degradation of *Deg1*∗-Sec62 is sensitive to lipid biosynthesis perturbation

**Figure 3:**
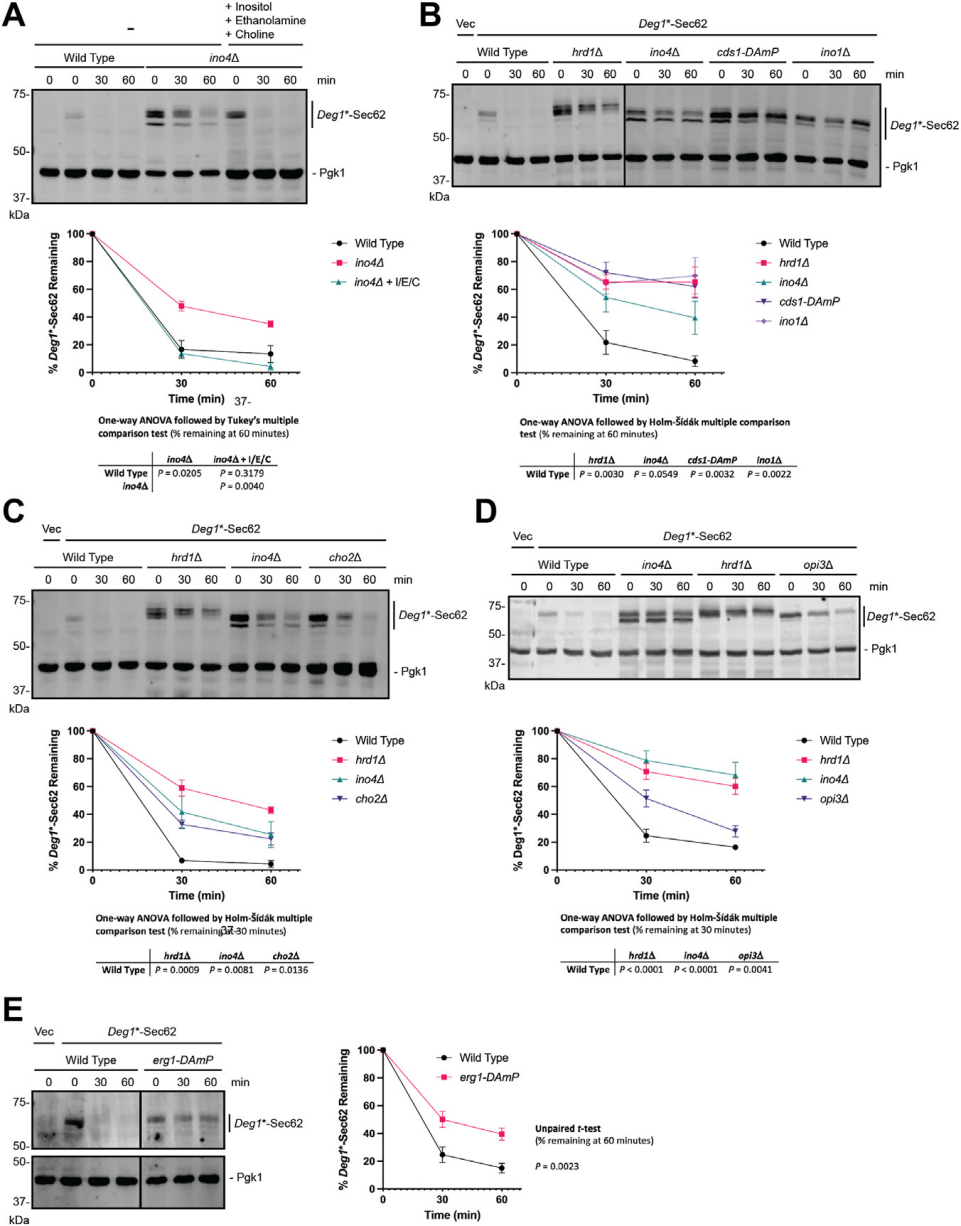
***Deg1*∗-Sec62 degradation is sensitive to perturbation of lipid biosynthesis.** Yeast of the indicated genotypes were transformed with a plasmid encoding *Deg1*∗-Sec62 or an empty vector and subjected to cycloheximide chase and western blot analysis to detect *Deg1*∗-Sec62 and Pgk1. Means of percent *Deg1*∗-Sec62 remaining for 3 to 5 biological replicates are plotted. Error bars represent the standard error of the mean. For the experiment depicted in (*A*), the final three lanes represent yeast supplemented with 500 μM inositol, 2 mM ethanolamine, and 2 mM choline from inoculation through cell harvest and cycloheximide chase. Means of percent *Deg1*∗-Sec62 remaining 60 min in (*A*) were evaluated by one-way ANOVA followed by Tukey’s multiple comparison test. Means of percent remaining at indicated times in (*B*–*D*) were evaluated by one-way ANOVA followed by Holm-Šídák multiple comparison tests (only pairs relative to wild-type yeast were compared). Means of percent remaining at 60 min in (*E*) were evaluated by an unpaired, two-tailed *t* test.

Supplementation of media with inositol, ethanolamine, and choline (lipid biosynthetic intermediates whose synthesis and uptake are mediated by targets of Ino2/Ino4 (30)) restored *Deg1*∗-Sec62 degradation in *ino4*Δ yeast (Fig. 3*A*). This is consistent with perturbed lipid biosynthesis causing the degradation defect. Ino2/Ino4 regulates expression of genes encoding enzymes mediating multiple branches of phospholipid biosynthesis. We analyzed *Deg1*∗-Sec62 degradation in yeast harboring deletions or hypomorphic alleles of four of these genes: *CDS1*, *INO1*, *CHO2*, and *OPI3*. Cds1 promotes the synthesis of cytidine diphosphate, a precursor of several membrane lipids, including phosphatidylinositol derivatives, cardiolipin, phosphatidylserine, phosphatidylethanolamine, and phosphatidylcholine (42). Ino1 catalyzes the conversion of glucose-6-phosphate to a precursor of inositol and is essential for *de novo* synthesis of phosphatidylinositol derivatives (43). Cho2 and Opi3 are required for *de novo* synthesis of phosphatidylcholine (44, 45). Mutation of *CDS1* or *INO1* strongly stabilized *Deg1*∗-Sec62 (Fig. 3*B*). Deletion of *CHO2* or *OPI3* also slowed *Deg1*∗-Sec62 turnover (Fig. 3, *C* and *D*).

*ERG1* encodes squalene epoxidase, which mediates an essential step in the biosynthesis of ergosterol, the primary sterol in fungal membranes (46). The ΔOD_595_ value for *erg1-DAmP* yeast in our screen was 0.078, just beyond the 0.079 cutoff. Perturbation of *ERG1*, which is not regulated by Ino2/Ino4, enhanced *Deg1*∗-Sec62 stability (Fig. 3*E*). Together, these results indicate *Deg1*∗-Sec62 degradation is broadly sensitive to perturbation in membrane lipid biosynthesis.

### *INO4* deletion causes a generalized ERAD defect

**Figure 4:**
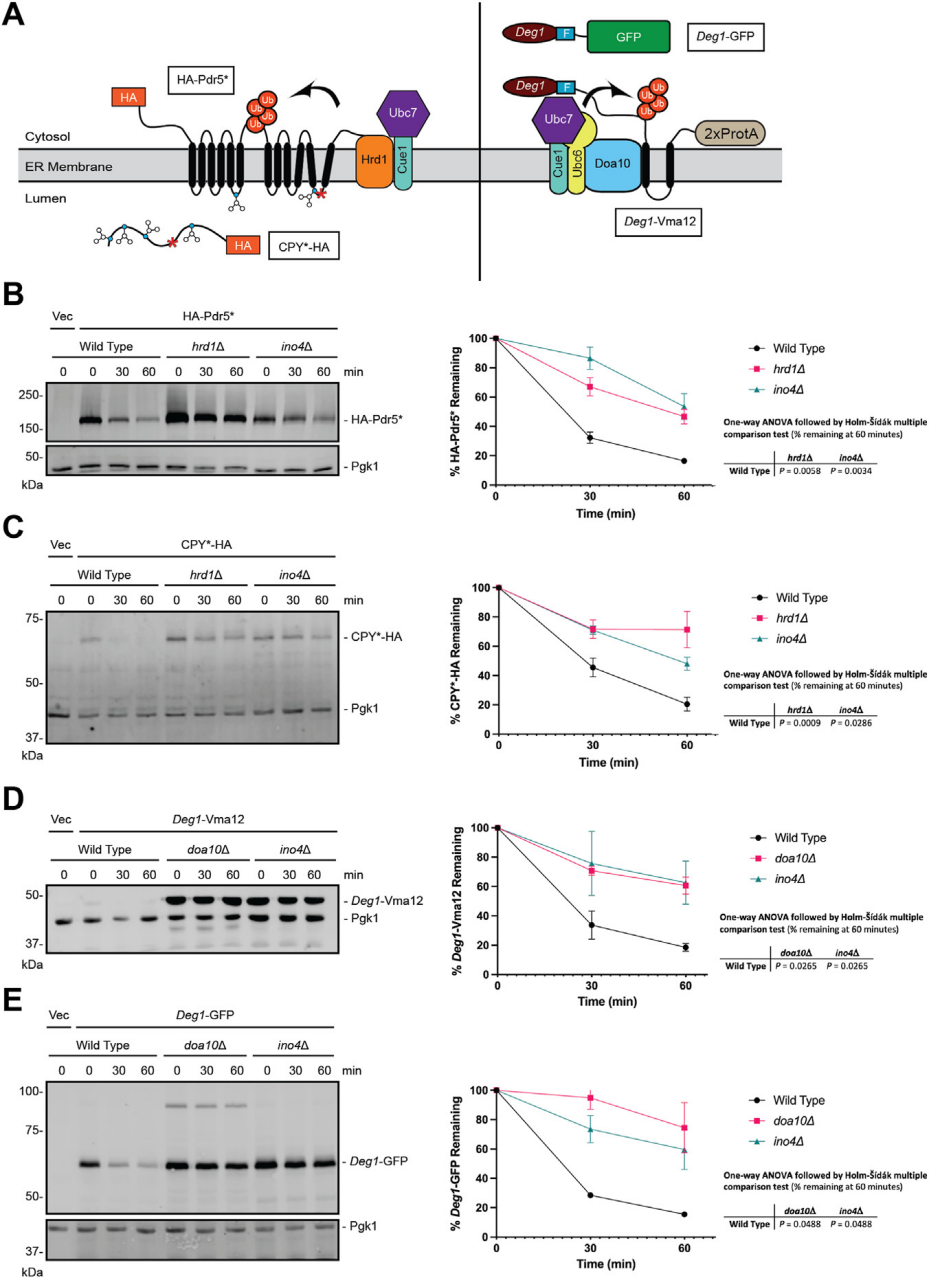
***INO4* deletion impairs ERAD of Hrd1 and Doa10 substrates.***A*, ERAD substrates of Hrd1 and Doa10 analyzed in this figure. *B*–*E*, yeast of the indicated genotypes were transformed with a plasmid encoding indicated ERAD substrates or an empty vector and subjected to cycloheximide chase and western blot analysis to detect the ERAD substrate and Pgk1. Means of percent ERAD substrate remaining for 3 to 6 biological replicates are plotted. Error bars represent standard error of the mean. Means of percentage of ERAD substrate remaining at 60 min were evaluated by one-way ANOVA followed by Holm-Šídák multiple comparison tests (only pairs relative to wild-type yeast were compared). F, Flag; ProtA, Protein A; Ub, ubiquitin.

**Figure 5:**
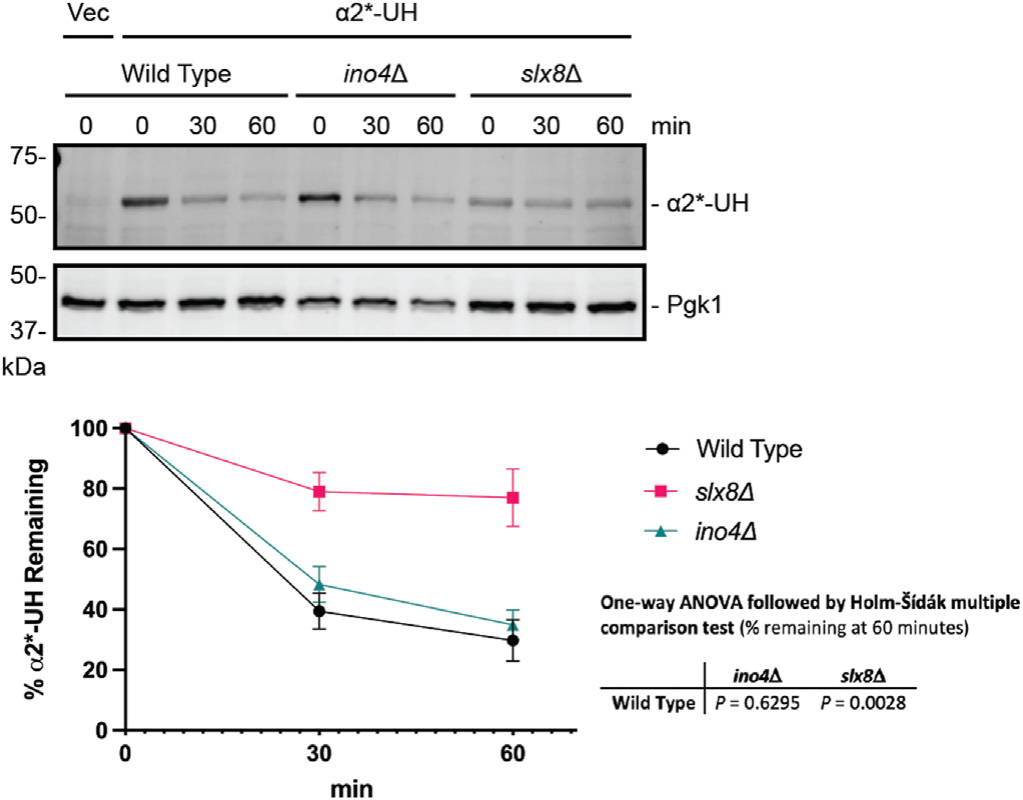
***INO4* deletion does not impair degradation of a soluble, nucleoplasmic substrate of soluble ubiquitin ligase Slx5/Slx8.** Yeast of the indicated genotypes were transformed with a plasmid encoding α2∗-Ura3-3HA (α2∗-UH) or an empty vector and subjected to cycloheximide chase and western blot analysis of α2∗-UH and Pgk1. Means of percent α2∗-UH remaining for four biological replicates are plotted. Error bars represent standard error of the mean. Means of percent α2∗-UH remaining at 60 min were evaluated by one-way ANOVA followed by Holm-Šídák multiple comparison tests (only pairs relative to wild-type yeast were compared).

We investigated the requirement of *INO4* for the degradation of a panel of Hrd1 and Doa10 ERAD substrates (Fig. 4*A*). *INO4* deletion stabilized an integral membrane Hrd1 substrate bearing an intramembrane degradation signal (HA-Pdr5∗, Fig. 4*B* (47)), a soluble, luminal Hrd1 substrate (CPY∗-HA, Fig. 4*C* (48)), an integral membrane Doa10 substrate bearing a cytosolic degron (*Deg1*-Vma12, Fig. 4*D* (18)), and a soluble, cytosolic Doa10 substrate (*Deg1*-GFP, Fig. 4*E* (49)). Thus, deletion of *INO4* broadly impairs protein degradation mediated by the ERAD ubiquitin ligases Hrd1 and Doa10. By contrast, degradation of a soluble, non-ER-associated, nucleoplasmic substrate (α2∗-Ura3-3HA) of a soluble ubiquitin ligase (Slx5/Slx8) (50) was unaffected by *INO4* deletion (Fig. 5).

**Figure 6:**
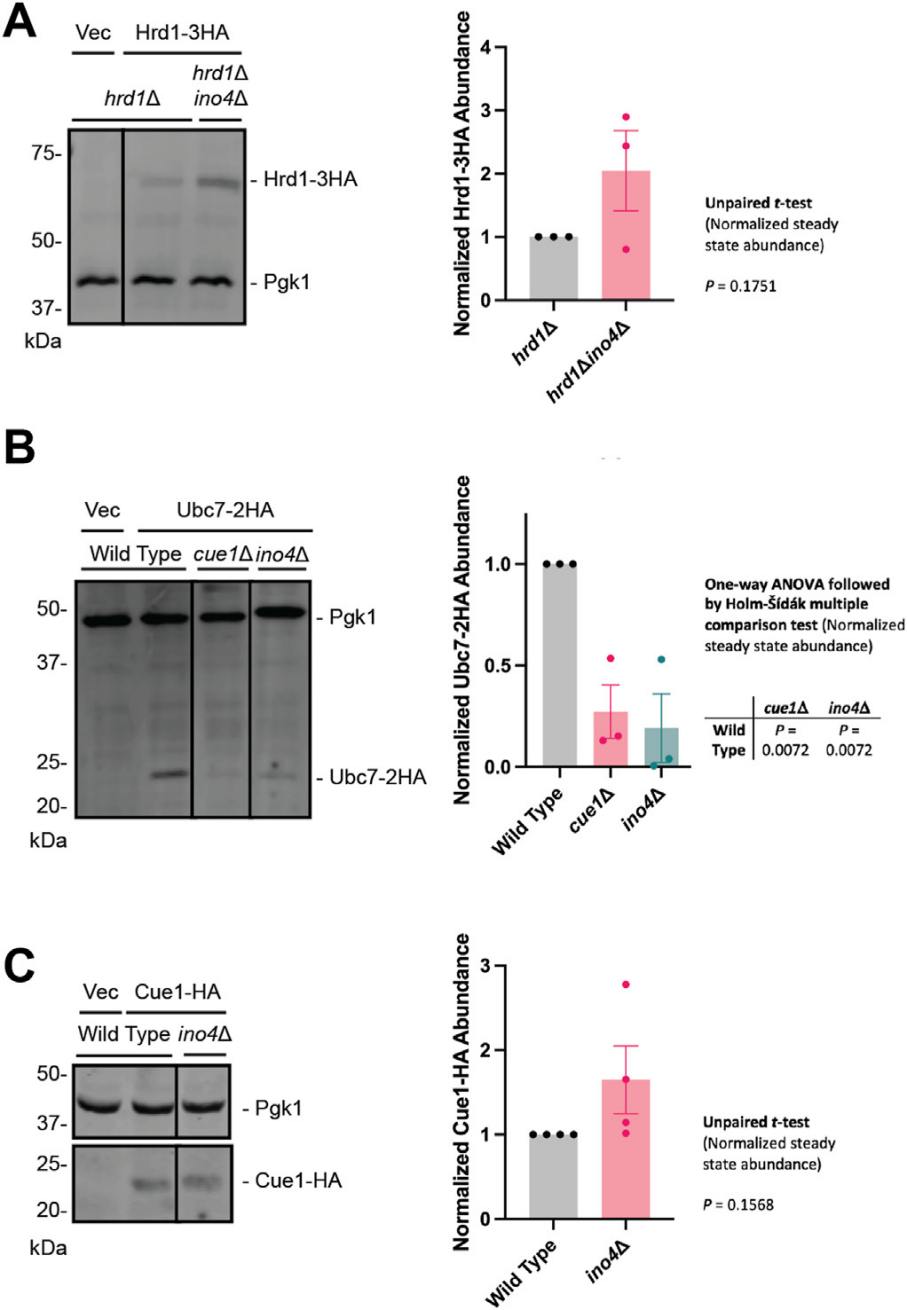
**Loss of *INO4* reduces Ubc7-2HA abundance.***Left*, Yeast of the indicated genotypes were transformed with a plasmid encoding Hrd1-3HA, Ubc7-2HA, Cue1-HA, or an empty vector, harvested, lysed, and subjected to anti-HA and anti-Pgk1 western blotting. Means of steady state abundance for 3 to 4 biological replicates are plotted. Error bars represent the standard error of the mean. Means in (*A* and *C*) were evaluated by an unpaired, two-tailed *t* test. Means in (*B*) were evaluated by one-way ANOVA followed by a Holm-Šídák multiple comparison test (only pairs relative to wild-type yeast were compared).

One potential mechanism by which *INO4* deletion compromises ERAD is through the reduction of the abundance of one or more components of the ubiquitylation machinery. We analyzed the abundance of plasmid-encoded Hrd1-3HA in yeast expressing or lacking *INO4*. Hrd1-3HA is functional *in vivo* (19). Hrd1-3HA abundance was elevated in *ino4*Δ yeast but not significantly (Fig. 6*A*). Both Doa10 and Hrd1 require the ubiquitin-conjugating enzyme Ubc7 and its membrane anchor Cue1. Under some circumstances, Hrd1 undergoes autoubiquitylation and degradation in a Ubc7-dependent manner (5, 51). Thus, impaired ERAD and increased Hrd1 abundance could be attributed to reduced abundance of Ubc7. We therefore assessed abundance of plasmid-encoded 2HA-tagged Ubc7 in wild-type yeast, yeast lacking Cue1, and yeast lacking Ino4. HA-tagging of Ubc7 does not abolish activity or ubiquitin ligase interaction (52, 53). Consistent with previous reports demonstrating *CUE1* deletion destabilizes Ubc7 (9, 53, 54), steady state abundance of Ubc7-2HA was significantly reduced in *cue1*Δ cells (Fig. 6*B*). Ubc7-2HA abundance was similarly reduced in *ino4*Δ yeast. We did not observe a difference in HA-tagged Cue1 steady state abundance in yeast lacking *INO4* (Fig. 6*C*), suggesting the reduction in Ubc7-2HA levels in *ino4*Δ may be independent of Cue1.

### *INO4* deletion does not cause a general translocation defect

**Figure 7:**
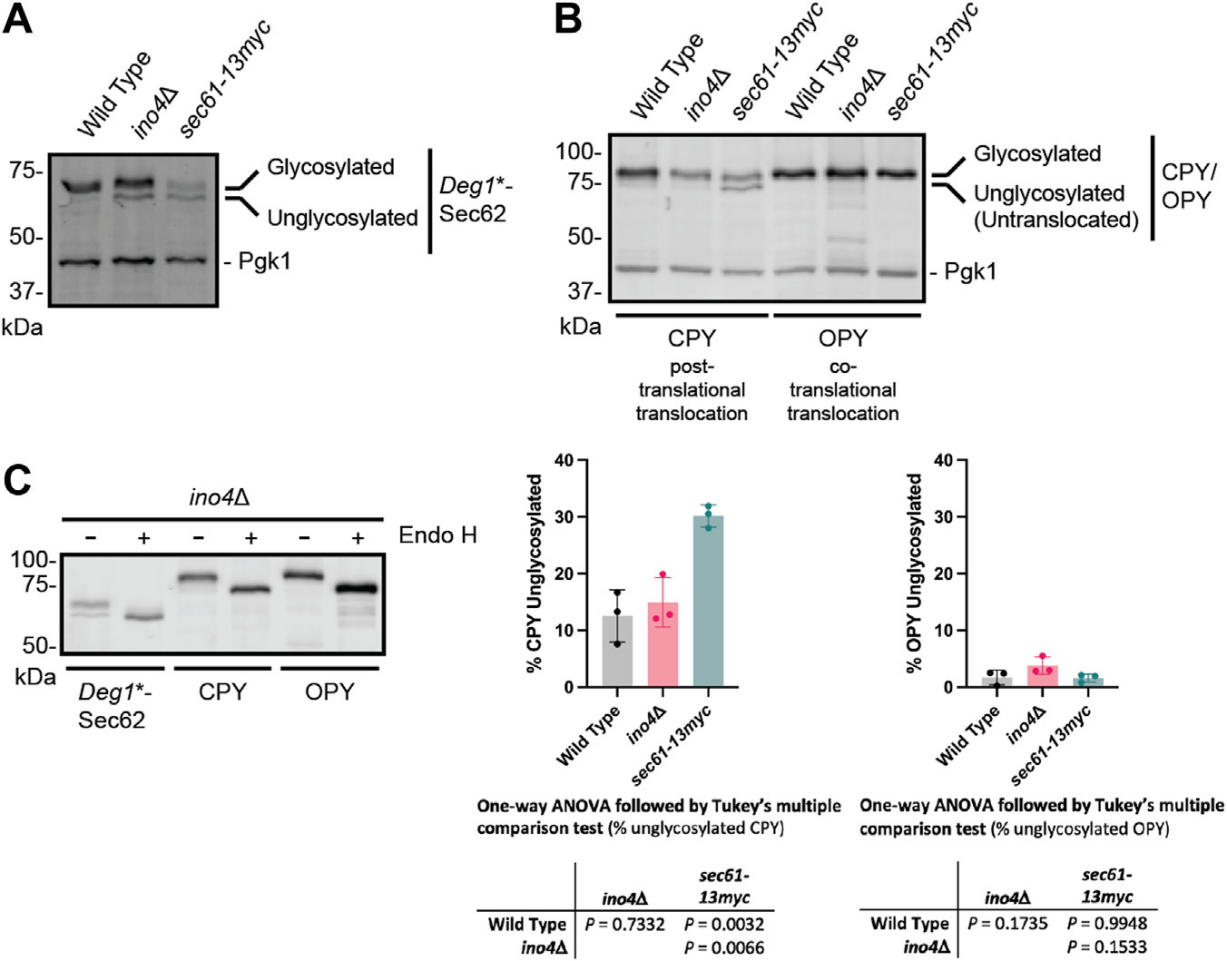
***INO4* deletion does not broadly impair translocation.***A*, electrophoretic migration of plasmid-encoded *Deg1*∗-Sec62 in yeast of the indicated genotypes was assessed by western blotting. *B*, *top*, Electrophoretic migration of plasmid-encoded CPY (a model post-translationally translocated protein) or OPY (a model co-translationally translocated protein) in yeast of the indicated genotypes was assessed by western blotting. *Bottom*, Means of the proportion of CPY or OPY that is unglycosylated (*i.e.*, untranslocated) for three biological replicates are plotted. Error bars represent standard error of the mean. Means were evaluated by one-way ANOVA followed by Tukey’s multiple comparison test. *C*, lysates from *ino4*Δ yeast expressing *Deg1*∗-Sec62, CPY, or OPY were incubated in the presence or absence of Endoglycosidase H (Endo H) prior to western blotting.

*Deg1*∗-Sec62 becomes glycosylated following aberrant post-translational translocation of its N-terminal tail (14). Deletions and hypomorphic alleles of *INO4* and several other lipid biosynthetic genes delay *Deg1*∗-Sec62 post-translational modification (Figure 2, Figure 3, and 7*A*). A potential contributor to impaired ERAD following perturbed lipid biosynthesis is globally delayed translocation, which might result in reduced ER localization of ERAD components. We analyzed translocation of a model post-translationally translocated protein (carboxypeptidase Y; CPY) and co-translationally translocated protein (a variant of CPY with the Ost1 signal sequence; OPY (55)). Upon ER entry, both CPY and OPY become *N*-glycosylated and display reduced electrophoretic mobility (55). While *INO4* deletion reduced post-translational modification of *Deg1*∗-Sec62, modification of CPY and OPY were unaffected (Fig. 7, *A* and *B*). By contrast, appendage of a 13myc epitope to the Sec61 translocon subunit impaired post-translational translocation of *Deg1*∗-Sec62 and CPY, as previously reported (15). Endoglycosidase H (Endo H) sensitivity of *Deg1*∗-Sec62, CPY, and OPY confirm *N*-glycosylation of these proteins in *ino4*Δ yeast (Fig. 7*C*). These results suggest dampened ERAD by compromised lipid biosynthesis is not due to a generalized translocation defect.

### Genetic perturbation of lipid biosynthesis sensitizes yeast to hygromycin B

**Figure 8:**
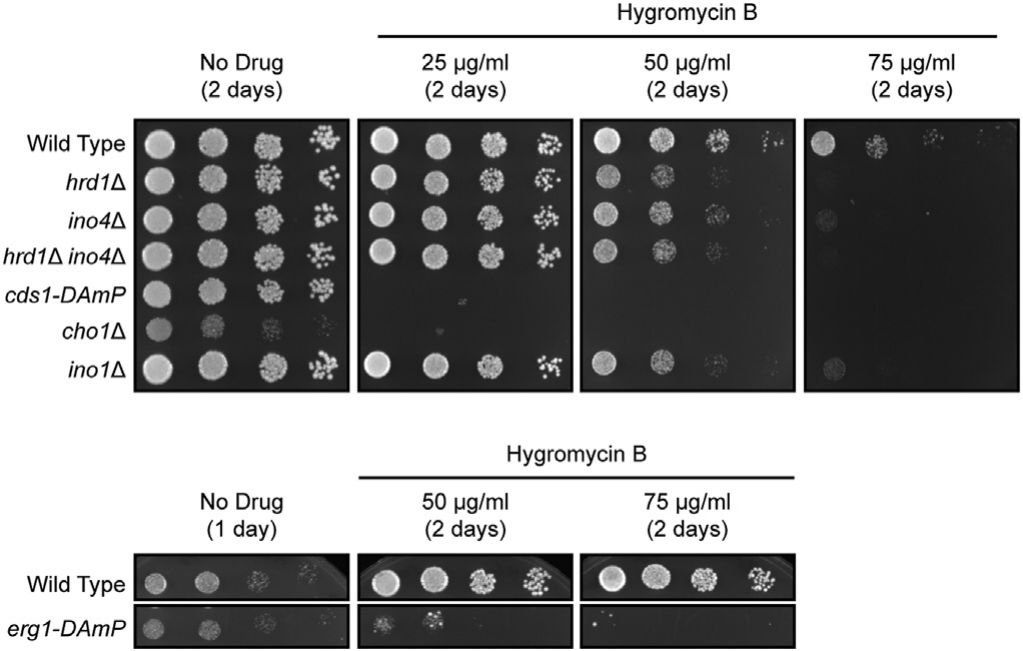
**Genetic perturbation of lipid biosynthesis sensitizes yeast to hygromycin B.** Yeast of the indicated genotypes were serially diluted and spotted onto rich yeast agar medium (YPD) with or without hygromycin B. Plates were incubated at 30 °C and imaged after 1 to 2 days. Experiments were performed in triplicate (*i.e.*, three biological replicates).

Given the impact of *INO4* deletion on the degradation of several model aberrant proteins, we predicted disruption of genes required for lipid biosynthesis would sensitize yeast to hygromycin B. Hygromycin B distorts the ribosome aminoacyl site, resulting in globally increased production of aberrant polypeptides (56, 57). We analyzed the growth of wild-type yeast and yeast possessing deletions or hypomorphic alleles of *HRD1*, *INO4*, *CDS1*, *CHO1* (required for phosphatidylserine, phosphatidylethanolamine, and phosphatidylcholine synthesis (30)), *INO1*, or *ERG1* in the absence or presence of hygromycin B (Fig. 8). As previously documented, *HRD1* deletion sensitized yeast to hygromycin B (23, 58, 59, 60). Likewise, all tested lipid biosynthesis mutants were hypersensitive to hygromycin B. *hrd1*Δ *ino4*Δ yeast did not exhibit enhanced sensitivity relative to *hrd1*Δ yeast. By contrast, impaired phospholipid biosynthesis did not markedly sensitize yeast to tunicamycin or elevated temperatures (Fig. S2, *A* and *B*). Sensitivity of *ino4*Δ, *cds1-DAmP*, *cho1*Δ, *ino1*Δ, and *erg1-DAmP* yeast to hygromycin B is consistent with a broad requirement of membrane lipid homeostasis for proteostasis.

## Discussion

In this study, we conducted a screen to identify genes required for the degradation of a model translocon-associated Hrd1 ERAD substrate. This screen revealed three novel genetic requirements for efficient *Deg1*∗-Sec62 degradation (*INO4*, *KAR3*, and *SET2*). We characterized the involvement of *INO4* and lipid biosynthetic enzymes in ERAD. *KAR3* was previously identified in a screen for genes required for the degradation of a Doa10 substrate (18). Further, GO analysis yielded enrichment of genes linked to sulfur metabolism. The impact of *KAR3* deletion on ERAD and the interplay between sulfur metabolism and ER homeostasis will be explored in subsequent studies.

Our screen revealed genes encoding two ERAD components (*HRD1* and *HRD3*) previously found to be required for efficient *Deg1*∗-Sec62 degradation and a proteasome assembly chaperone (*UMP1*). It did not reveal novel genes encoding proteins likely to be directly involved in the degradation of ER translocon-associated proteins. While such factors may exist and may have failed to have been recovered in this screen, it is possible that ERAD of translocon-associated proteins may be more austere than other ER protein degradation mechanisms. We previously showed loss of several genes required for recognition and ER extraction of Hrd1-dependent turnover of luminal and transmembrane substrates (*YOS9*, *USA1*, *DER1*, and *DFM1*) has minimal impact on *Deg1*∗-Sec62 stability (14). ERAD of translocon-clogging proteins may be mediated by the skeleton crew of Hrd1, Hrd3, Cue1, and Ubc7. How such translocon-associated proteins are recognized remains to be determined. Hrd1 has been proposed to directly recognize misfolded membrane proteins through interactions with its transmembrane segments (19). Hrd1 may similarly directly bind clogged translocons and ubiquitylate persistently engaged proteins. Consistent with this model, yeast and mammalian Hrd1 homologs have been found in complexes with the ER translocon (61, 62).

Neither *INO2* nor *INO4* has, to our knowledge, been identified in previous yeast screens for genetic requirements of ER protein degradation (*e.g.*, (4, 6, 18, 28, 63, 64, 65)). This is likely related to the fact that yeast lacking *INO2* or *INO4* exhibit dampened growth in minimal media (Fig. S2*C*), commonly used in growth reporter-based genetic screens. Our identification of *INO4* highlights the power of our screen to reveal genetic requirements for degradation.

Our results suggest ERAD is broadly sensitive to perturbations in lipid homeostasis. Deletion of either gene encoding members of the Ino2/Ino4 transcriptional regulator stabilized the translocon quality control substrate *Deg1*∗-Sec62 to a similar extent as *HRD1* deletion. Stabilization is likely due to altered membrane lipid composition, as supplementation with lipid biosynthetic intermediates rescued the *Deg1*∗-Sec62 degradation defect of *ino4*Δ yeast. Further, disruption of Ino2/Ino4-regulated genes encoding lipid biosynthetic enzymes impeded degradation, as did perturbation of a gene encoding an enzyme required for sterol biosynthesis (*ERG1*, not regulated by Ino2/Ino4). *INO4* deletion also stabilized a panel of model soluble and transmembrane targets of two ERAD ubiquitin ligases, Hrd1 and Doa10. By contrast, degradation of a soluble, nucleoplasmic substrate of a soluble ubiquitin ligase was unaffected by *INO4* deletion. Deletion of *INO4* and mutation of genes required for different aspects of lipid biosynthesis sensitized yeast to hygromycin B, which is expected to increase the cellular burden of misfolded proteins. Future studies should be conducted to assess the impact of perturbed lipid biosynthesis on the degradation of regulated (*i.e.*, non-quality control) substrates of Hrd1 and Doa10 (*e.g.*, Hmg2 and Erg1, respectively (4, 66)) and substrates of ubiquitin ligases in other cellular compartments (*e.g.*, the inner nuclear membrane Asi complex).

How does disrupted lipid composition impair ER protein quality control? As a consequence of altered membrane fluidity or protein-lipid interactions, perturbation of membrane lipid composition may change the abundance, structure, membrane integration or docking, or localization of substrate recognition or ubiquitylation machinery. Our results suggest the ubiquitin-conjugating enzyme Ubc7 (which is required for both Hrd1-and Doa10-dependent ERAD) is present in reduced abundance in *ino4*Δ yeast. Ubc7 is anchored to the ER membrane – and stabilized – by Cue1 (9, 53, 54). Recent work demonstrated that altered membrane phosphatidylcholine abundance modestly destabilizes Cue1 (67); however, we did not observe a decrease in Cue1-HA levels in *ino4*Δ yeast. Reduced Ubc7 abundance (*via* either accelerated degradation or dampened synthesis) might contribute to broad ERAD impairment. We sought to determine if *UBC7* overexpression rescues the degradation defect in *ino4*Δ yeast. However, efforts to culture *ino4*Δ yeast overexpressing *UBC7* and harboring protein quality control substrates were unsuccessful (unpublished observations). We note that our analysis of the impact of *INO4* deletion on Ubc7 abundance was conducted using plasmid-encoded, epitope-tagged Ubc7 in yeast that also expressed endogenous, untagged *UBC7*; these results may not reflect the effects of altered lipid synthesis on chromosome-encoded, untagged Ubc7. Future experiments will be conducted to assess if, and how, perturbed membrane composition alters expression of ERAD components, and if altered ERAD machinery abundance is sufficient to explain the impact of altered lipid composition on protein degradation.

*Deg1*∗-Sec62 is glycosylated upon its aberrant translocon engagement (14). This modification was delayed in yeast lacking several genes encoding lipid biosynthetic enzymes, consistent with dampened translocation rate. While lipid bilayer stress might slow ER import of one or more proteins required for ERAD, our results indicate *INO4* deletion does not cause a generalized translocation block. It is also conceivable that altered membrane lipid composition impedes ERAD substrate *retro*translocation (movement from the ER to the cytosol for proteasomal degradation). However, impaired retrotranslocation cannot explain the totality of the impact of *INO4* deletion, as a model soluble, cytosolic Doa10 substrate (*Deg1*-GFP) was stabilized in *ino4*Δ yeast.

In contrast to impaired degradation of the Doa10 substrates evaluated in this study, lipid bilayer stress caused by phosphatidylcholine depletion (*i.e.*, *OPI3* deletion) accelerates the degradation of Doa10 substrate Sbh1 (67). Sbh1 is atypically degraded by Doa10, as its turnover occurs independent of cytosolic lysine residues (67). Thus, Doa10 activity *per se* may not be impaired by alterations in lipid composition. Comparing the mechanism of canonical *versus* atypical Doa10 degradation mechanisms may reveal molecular factors that are differentially sensitive to membrane lipid composition.

In a previous study, we found *Deg1*∗-Sec62 degradation occurs with wild-type kinetics in the context of inositol depletion (68). Data from the present study appear to contradict this observation, which we reproduced (Fig. S3). We speculate this discrepancy reflects differences in duration of lipid perturbation. In our earlier study, yeast experienced acute (5 h) inositol restriction, whereas yeast in the present study were subjected to genetic (*i.e.*, long-term) perturbations in lipid biosynthesis.

This work builds on expanding literature linking lipid homeostasis to ER proteostasis. Lipid bilayer stress activates the yeast and mammalian ER unfolded protein response (UPR) *via* a distinct mechanism than activation by unfolded proteins (69, 70, 71, 72, 73, 74, 75). The protein homeostatic machinery induced by the UPR buffers the toxic effects of disrupted lipid homeostasis (70), and genes required for lipid biosynthesis are synthetically lethal with those encoding UPR mediators (70, 76). Further, accumulation of misfolded ER proteins (*i.e.*, ER stress) promotes proteolytic activation of the mammalian SREBP transcription factors, which regulate lipid metabolism (77, 78). In addition to stimulating expression of genes required for lipid biosynthesis, cleaved SREBP1 promotes UPR signaling (79).

Consistent with the broad conservation of the relationship between lipid and protein homeostasis, several instances of altered lipid composition impacting protein degradation have been reported. For example, inhibition of long-chain acyl-coA synthetases impairs glycan trimming, ER extraction, and degradation of a subset of glycosylated substrates of the mammalian HRD1 ubiquitin ligase (80). Degradation of yeast CPY∗ is sensitive to *CHO2* or *OPI3* deletion (70) and, more modestly, to mutations that impair lipid droplet formation (81). Very recently, an elevated abundance of long-chain ceramides was shown to impede retrotranslocation and degradation of several ERAD substrates (82). Local lipid metabolism regulates the turnover of mammalian nucleo-cytoskeletal linker Sun2 at the inner nuclear membrane (83). Yeast and mammalian homologs of Hrd1 and Doa10 promote feedback-regulated degradation of sterol-biosynthetic enzymes as well as the turnover of proteins implicated in triacylglycerol and low-density-lipoprotein metabolism (4, 66, 84, 85, 86, 87). Together with these studies, our work strongly suggests a profound interdependence between lipid dynamics and ER protein degradation.

Perturbed membrane lipid composition has been implicated in multiple diseases, including non-alcoholic fatty liver disease (31, 32), obesity and type II diabetes (33), muscular dystrophy (34), and cardiomyopathies (35). Phospholipid metabolism may also be altered in Alzheimer’s disease (36, 88, 89, 90, 91). Our results suggest alterations in lipid profiles associated with these disease states are likely to impair ERAD. Further investigation of the relationship between lipid and protein homeostasis may inform improved understanding and treatment of diseases associated with disruptions in cellular lipid dynamics.

## Experimental procedures

### Yeast and plasmid methods

Yeast were cultured at 30 °C in standard rich (yeast extract-peptone-dextrose, YPD) or minimal (synthetic defined, SD) growth medium (92). Plasmids were introduced into yeast *via* lithium acetate transformation (92). See Table 3 for yeast strains used in this study. See Table 4 for plasmids used in this study.

**Table 3:** Yeast strains used in this study

Name	Genotype	Source
VJY6	*MATa his3-*Δ*200 leu2-3112 ura3-52 lys2-801 trp1-1 gal2*	(107) (alias MHY500)
VJY10	*MATa his3-*Δ*200 leu2-3112 ura3-52 lys2-801 trp1-1 gal2 hrd1*Δ::*kanMX4*	(37)
VJY102	*MATa his3*Δ*1 leu2*Δ*0 met15*Δ*0 ura3*Δ*0 doa10*Δ::*kanMX4*	(38)
VJY146	*MATα ade2 his3 leu2 ura3 trp1 can1-100 ydj1-2::HIS3 ydj1-151::LEU2 hlj1::TRP1*	(16) (alias SM4949)
VJY324	*MATa his3*Δ*1 leu2*Δ*0 met15*Δ*0 ura3*Δ*0 cue1*Δ::*kanMX4*	(38)
VJY338	*MATα can1*Δ::*STE2pr-LEU2 lyp1*Δ *ura3*Δ*0 leu2*Δ*0 his3*Δ*1*	(94) (alias Y7039)
VJY355	*MATα can1*Δ::*STE2pr-LEU2 lyp1*Δ *ura3*Δ*0 leu2*Δ*0 his3*Δ*1 doa10*Δ::*P*_*GAL4*_*-DSPH*:*natmx4*	This study
VJY474	*MATa his3*Δ*1 leu2*Δ*0 met15*Δ*0 ura3*Δ*0 ino4*Δ::*kanMX4*	(38)
VJY476	*MATa his3*Δ*1 leu2*Δ*0 met15*Δ*0 ura3*Δ*0*	(38) (alias BY4741)
VJY478	*MATα his3*Δ*1 leu2*Δ*0 lys2*Δ*0 ura3*Δ*0 hrd1*Δ::*kanMX4*	(38)
VJY489	*MATα ade2 his3 leu2 ura3 trp1 can1-100*	(16) (alias SM4947)
VJY511	*MATa his3*Δ*1 leu2*Δ*0 met15*Δ*0 ura3*Δ*0 hrd1*Δ::*kanMX4*	(38)
VJY568	*MATa his3*Δ*1 leu2*Δ*0 met15*Δ*0 ura3*Δ*0 set2*Δ::*kanMX4*	(38)
VJY659	*MATa his3*Δ*1 leu2*Δ*0 met15*Δ*0 ura3*Δ*0 slx8*Δ::*kanMX4*	(38)
VJY736	*MATa his3*Δ*1 leu2*Δ*0 met15*Δ*0 ura3*Δ*0 ino2*Δ::*kanMX4*	(38)
VJY753	*MATa his3*Δ*1 leu2*Δ*0 met15*Δ*0 ura3*Δ*0 erg1-DAmP*:*kanMX4*	(95)
VJY780	*MATa his3*Δ*1 leu2*Δ*0 met15*Δ*0 ura3*Δ*0 kar3*Δ::*kanMX4*	(38)
VJY951	*MATa his3*Δ*1 leu2*Δ*0 met15*Δ*0 ura3*Δ*0 hrd1*Δ::*kanMX4 ino4*Δ::*kanMX4*	This study
VJY963	*MATa his3*Δ*1 leu2*Δ*0 met15*Δ*0 ura3*Δ*0 ino1*Δ::*kanMX4*	(38)
VJY964	*MATa his3*Δ*1 leu2*Δ*0 met15*Δ*0 ura3*Δ*0 cho1*Δ::*kanMX4*	(38)
VJY965	*MATa his3*Δ*1 leu2*Δ*0 met15*Δ*0 ura3*Δ*0 cds1-DAmP:kanMX4*	(95)
VJY979	*MATa his3*Δ*1 leu2*Δ*0 met15*Δ*0 ura3*Δ*0 cho2*Δ::*kanMX4*	(38)
VJY1071	*MATa his3*Δ*1 leu2*Δ*0 met15*Δ*0 ura3*Δ*0 opi3*Δ::*kanMX4*	(38)

**Table 4:** Plasmids used in this study

Name	Alias	Yeast selection marker	Yeast plasmid type	Description	Source
pHA-Pdr5∗	pVJ1; pRH2312	*HIS3*	CEN	HA-tagged Pdr5∗; Pdr5∗ = C1427Y	(19)
YCp50-*P*_*PRC1*_-CPY∗-HA	pVJ2; pDN431	*URA3*	CEN	HA-tagged CPY∗ driven by native promoter; CPY∗ = G255R	(108)
pRS313	pVJ26	*HIS3*	CEN	Empty vector	(109)
pRS316	pVJ27	*URA3*	CEN	Empty vector	(109)
pRS315	pVJ40	*LEU2*	CEN	Empty vector	(109)
p414-*P*_*MET25*_	pVJ121	*TRP1*	CEN	Empty vector with *MET25* promoter	(110)
p416-*Deg1*-GFP2	pVJ205; pUL28	*URA3*	CEN	*Deg1* fused to two copies of GFP	(49)
p416-*P*_*MET25*_-*Deg1*∗-Sec62-2xProtA	pVJ317	*URA3*	CEN	*Deg1∗*-Flag-Sec62-2xProtA (“*Deg1*∗-Sec62”) driven by *MET25* promoter; *Deg1*∗ = F18S, I22T	(14)
p416-*P*_*GPD*_-*Deg1*-Vma12-2xProtA	pVJ343	*URA3*	CEN	*Deg1*-Flag-Vma12-2xProtA (“*Deg1*-Vma12”) driven by *TDH3* (*GPD*) promoter	(68)
p414-*P*_*GAL4*_-*Deg1*-Sec62-ProtA-His3	pVJ477	*TRP1*	CEN	*Deg1*-Flag-Sec62-2xProtA-His3 (*“Deg1*-Sec62-His3”) driven by *GAL4* promoter	(37)
p414-*P*_*GAL4*_-*Deg1*-Sec62-2xProtA-His3:natmx4	pVJ490	*TRP1, natMX4*	CEN	Source of *P*_*GAL4*_*-Deg1-Sec62-His3:natmX4* cassette to generate query strain for screen; *Deg1-Flag-Sec62-2xProtA-His3* (*“Deg1*-Sec62-His3”) driven by *GAL4* promoter; *natMX4* is a distinct gene with its own promoter and terminator	This study
pRS313-*ADH*-Ubc7-2HA	pVJ520; STK06-2-5	*HIS3*	CEN	2HA-tagged Ubc7 driven by *ADH* promoter	(111)
pRS314-UPRE-GFP	pVJ552	*TRP1*	CEN	GFP driven by Unfolded Protein Response Element (UPRE)	(112)
pRS316-GPD-CPY-2xProtA	pVJ576	*URA3*	CEN	CPY-2xProtA driven by *GPD* promoter	(15)
pRS316-GPD-OPY-2xProtA	pVJ578	*URA3*	CEN	OPY-2xProtA driven by *GPD* promoter OPY is CPY with the CPY signal sequence replaced with Ost1 signal sequence (55)	(15)
pJM130-MATα2∗-URA3-3HA	pVJ656	*LEU2*	CEN	MATα2 (a substrate of both Doa10 and Slx5/Slx8 ubiquitin ligases) possessing I4T and L10S mutations (which render substrate degradation primarily dependent on Slx5/Slx8) and C-terminal appendage of Ura3 and 3HA epitope	(50)
pRS315-Cue1-HA	pVJ667; pGT181	*LEU2*	CEN	HA-tagged Cue1	(67)

To generate pVJ490 (a plasmid containing *P*_*GAL4*_*-DEG1-SEC62-HIS3* and *natMX4* as independent genes that could be amplified as a single PCR product for genomic integration), a 1314-bp EagI fragment containing *natMX4* from pAG25 (alias pVJ132) (93) was inserted into the EagI site of pVJ477 (37), which possessed *P*_*GAL4*_*-DEG1-SEC62-HIS3*. The orientation of the *natMX4* fragment was confirmed by NcoI digestion.

To generate the query strain VJY355 for Synthetic Genetic Array (SGA) analysis (38), a cassette containing *P*_*GAL4*_*-DEG1-SEC62-HIS3* and *natMX4* flanked by 50 bp of DNA homologous to sequence upstream and downstream of the *DOA10* open reading frame was PCR-amplified from pVJ490 using primers VJR264 and VJR265 (see Table 5 for primers used in this study). This PCR product was introduced to Y7039 (haploid MATα query strain; alias VJY338 (94)), and integration was confirmed by three-primer PCR at the 5′ and 3′ ends of the *doa10*Δ::*P*_*GAL4*_*-DEG1-SEC62-HIS3*:*natMX4* locus using primers VJR46, VJR82, and VJR260 (5′ end) and VJR11, VJR107, and VJR249 (3′ end). To confirm integration of the cassette at a single locus, VJY355 was mated with nourseothricin-sensitive MATa haploid yeast. Sporulation of the mated diploid was induced, and 2:2 segregation of nourseothricin resistance:sensitivity was observed, consistent with integration of the *P*_*GAL4*_*-DEG1-SEC62-HIS3*:*natMX4* cassette at a single locus.

**Table 5:** Primers used in this study

Purpose	Primer #	Sequence	Product sizes
Amplify *P*_*GAL4*_*-DEG1-SEC62-HIS3*:*natMX4* cassette for integration at *DOA10* locus in SGA query strain from pVJ490	VJR264	5′ TAGCCAAGAGTACCACTAATTGAATCAAAGAGACTAGAAGTGTGAAAGTCcgcgcaattaaccctcacta 3′	4338 bp
	VJR265	5′ TATATGTAAATATGCTAGCATTCATTTTAAATGTAAGGAAGAAAACGCCTggcggcgttagtatcgaat 3′	

Confirm integration of *P*_*GAL4*_*-DEG1-SEC62-HIS3*:*natMX4* cassette at *DOA10* locus (5′ end)	VJR46	5′ CATGGTACCGAGCTCCTTGT 3′	WT *DOA10*–842 bp *doa10*Δ::*P*_*GAL4*_*-DEG1-SEC62-HIS3*:*natMX4* – 1073 bp
	VJR82	5′ AATCGGTAGCGCGTATGACT 3′	
	VJR260	5′ CGTGGTTAATTCTGGAGTTGC 3′	

Confirm integration of *P*_*GAL4*_*-DEG1-SEC62-HIS3*:*natMX4* cassette at *DOA10* locus (3′ end)	VJR11	5′ ATTCCCAACATGGACAAGGA 3′	WT *DOA10*–550 bp *doa10*Δ::*P*_*GAL4*_*-DEG1-SEC62-HIS3*:*natMX4* – 746 bp
	VJR107	5′ GTTTGGGATGAGGGCAGAG 3′	
	VJR249	5′ ACCTCTGGCTGGAGGTCAC 3′	

Genotyping *HRD1* locus (*HRD1versushrd1*Δ::*kanMX4*)	VJR70	5′ TGCAAAAAGGAAACGCTTGT 3′	WT *HRD1* – 780 bp *hrd1*Δ::*kanMX4* – 987 bp
	VJR163	5′ ATTGGCCATTAGAGGTGACG 3′	
	VJR259 (Alias kanB)	5′ CTGCAGCGAGGAGCCGTAAT 3′	

Genotyping *INO4* locus (*INO4versusino4*Δ::*kanMX4*)	VJR371	5′ GAAAAGGGTTGCAGTTAAGCA 3′	WT *INO4* – 450 bp *ino4*Δ::*kanMX4* – 655 bp
	VJR372	5′ TCTTCTTAGACCTGCGTTTCC 3′	
	VJR259 (Alias kanB)	5′ CTGCAGCGAGGAGCCGTAAT 3′	

To generate VJY951 (*hrd1*Δ::*kanMX4 ino4*Δ::*kanMX4*), MATα *hrd1*Δ::*kanMX4* yeast (VJY478) were mated with MATa *ino4*Δ::*kanMX4* yeast (VJY474). Mated *HRD1*/*hrd1*Δ::*kanMX4 INO4*/*ino4*Δ::*kanMX4* heterozygous diploids were induced to undergo sporulation, and spores were separated by microdissection. Candidate double mutant yeast were selected on the basis of 2:2 segregation of G418 resistance:sensitivity, and *HRD1* and *INO4* genotypes were verified by PCR using primers VJR70, VJR163, and VJR259 (to distinguish *HRD1* and *hrd1*Δ::*kanMX4*) and primers VJR371, VJR372, and VJR259 (to distinguish *INO4* and *ino4*Δ::*kanMX4*).

For supplementation experiments, yeast was cultured (from inoculation until cell harvest and cycloheximide chase) in media containing 500 μM inositol, 2 mM ethanolamine, and 2 mM choline. For the inositol limitation experiment, cells were cultured to mid-exponential growth in a medium containing inositol, washed six times in an inositol-free medium (prepared using yeast nitrogen base without amino acids and inositol), and incubated in an inositol-free medium for 5 h. Serial dilution growth assays were performed as described (37). With the exception of the genome-wide screen and the experiment depicted in Fig. S3, all experiments were performed 3 to 6 times, as indicated in figure legends.

### Screening of yeast deletion and hypomorphic allele collections

Screening of the yeast genome was performed as described in (38). VJY355 (MATα *his3*Δ*1* query strain possessing *P*_*GAL4*_*-DEG1-SEC62-HIS3* and *natMX4* at the *DOA10* locus) was mated with the haploid yeast MATa *his3*Δ*1* knockout and DAmP (Decreased Abundance by mRNA Perturbation) libraries of non-essential and essential genes, respectively (95, 96, 97). Each 96-well plate of the yeast knockout and DAmP collections includes a blank well (no yeast); *hrd1*Δ yeast were spiked into the blank well of each plate as a positive control for *Deg1*-Sec62-His3 stabilization. Following serial transfer using 96-prong pinners and culture of yeast on a series of selective media, a library of haploid MATa yeast expressing *Deg1*-Sec62-His3 and possessing knockout or hypomorphic alleles of each gene represented in the knockout and DAmP collections was generated. These yeast were transferred to 96-well plates possessing synthetic complete media and cultured for 48 h at 30 °C. 40 μl of each culture were transferred to 96-well plates containing 160 μl of selective media lacking histidine. The OD_595_ for each strain was recorded at the beginning and end of an 11-h incubation period at 30 °C using an iMark Microplate Absorbance Reader (Bio-Rad). A detailed outline of the SGA procedure is included in Table 1.

### Cycloheximide chase

Cycloheximide chase experiments were performed as previously described (98). Briefly, mid-exponential phase yeast cultured at 30 °C were concentrated to 2.5 OD_600_ units/ml in fresh synthetic defined medium and maintained at 30 °C. Cycloheximide was added to each culture (final concentration 250 μg/ml). 2.4-OD_600_ aliquots were harvested immediately after cycloheximide addition and at indicated time points and were added to stop mix containing sodium azide (final concentration 10 mM) and bovine serum albumin (final concentration 0.25 mg/ml). Samples were maintained on ice until the end of the chase, at which point all yeast were lysed.

### Cell lysis and endoglycosidase H treatment

Unless otherwise indicated, yeast were lysed using the alkaline lysis method, as previously described (37, 99). 2.4 to 2.5 OD_600_ units were harvested and suspended in 200 μl of 0.1 M NaOH, followed by incubation at room temperature for 5 min and pelleting by centrifugation. Pellets were resuspended in 1× Laemmli sample buffer and boiled at 100 °C for 5 min. Insoluble material was pelleted by high-speed centrifugation, and the soluble fraction (supernatant) was retained for electrophoresis. Endoglycosidase H (Endo H; New England Biolabs) treatment was conducted as described (100).

For analysis of HA-Pdr5∗ degradation (Fig. 4*B*), yeast were lysed using a trichloroacetic acid (TCA) lysis procedure as previously described (101). 2.4 OD_600_ units of yeast were harvested and suspended in 0.26 M NaOH and 0.13 M β-mercaptoethanol, followed by incubation on ice for 15 min. TCA (final concentration 5%) was added to cell suspensions to precipitate proteins, followed by centrifugation at 4 °C. Pellets were resuspended in TCA sample buffer (3.5% SDS, 0.5 M DTT, 80 mM Tris, 8 mM EDTA, 15% glycerol, 0.1 mg/ml bromophenol blue) and heated to 37 °C for 30 min. Insoluble material was pelleted by centrifugation (18,000*g* for 1 min), and the soluble fraction (supernatant) was retained for analysis by SDS-PAGE.

### Western blotting

Following separation by SDS-PAGE, proteins were transferred to polyvinylidene difluoride (PVDF) membrane *via* wet transfer at 20 V for 1 h at 4 °C. Membranes were blocked in 5% skim milk suspended in Tris-buffered saline (TBS; 50 mM Tris, 150 mM NaCl) at room temperature for 1 h or at 4 °C overnight. Antibody incubations were performed in 1% skim milk suspended in TBS with 1% Tween 20 (TBS/T) for 1 h at room temperature followed by three 5-min washes in TBS/T. The following primary antibody dilutions were used: mouse anti-HA.11 (Clone 16B12; BioLegend) at 1:1000; mouse anti-GFP (Clone JL-8; Clontech) at 1:1000; and mouse anti-Pgk1 (Clone 22C5D8; LifeTechnologies) at 1:20,000 to 1:40,000. Mouse primary antibodies were followed by incubation with AlexaFluor-680-conjugated rabbit anti-mouse secondary antibody (LifeTechnologies) at 1:20,000 to 1:40,000. Rabbit primary antibodies were followed by incubation with DyLight-800-conjugated goat anti-rabbit secondary antibody (Invitrogen). *Deg1*∗-Sec62, *Deg1*-Vma12, CPY, and OPY possess two copies of *Staphylococcus aureus* Protein A epitope, which interacts non-specifically with mammalian immunoglobulins (102) and were directly detected using the AlexaFluor-680-conjugated rabbit anti-mouse antibody. PVDF membranes were imaged with the Odyssey CLx IR Imaging System (Li-Cor). Protein abundance was determined using ImageStudio software (Li-Cor). Total fluorescence intensity for an area encompassing a protein was determined. Background intensity was extrapolated from the average fluorescence intensity of pixels near the protein and subtracted from total fluorescence intensity to yield an adjusted fluorescence intensity. Ratios of adjusted signal intensities for proteins of interest and loading control proteins were determined to compare samples in a given experiment.

### Statistical analysis

All data were analyzed using GraphPad Prism (version 9.5). Statistical tests employed are described in relevant figure legends. Calculated *p*-values are included in relevant figures.

## Data availability

All data are contained with the manuscript or associated Supplementary files.

## Supporting information

This article contains supporting information (14, 68, 72, 100, 103, 104, 105).

## Conflict of interest

The authors declare that they have no conflicts of interest with the contents of this article.
