# Supplementary material for: Lipid biosynthesis perturbation impairs endoplasmic reticulum–associated degradation
Source: J Biol Chem. 2023 Jun 17;299(8):104939. doi: 10.1016/j.jbc.2023.104939 (PMC10372827; doi:10.1016/j.jbc.2023.104939)
Supplement: Supporting information [file mmc2.docx]

**SUPPORTING FIGURES**

**
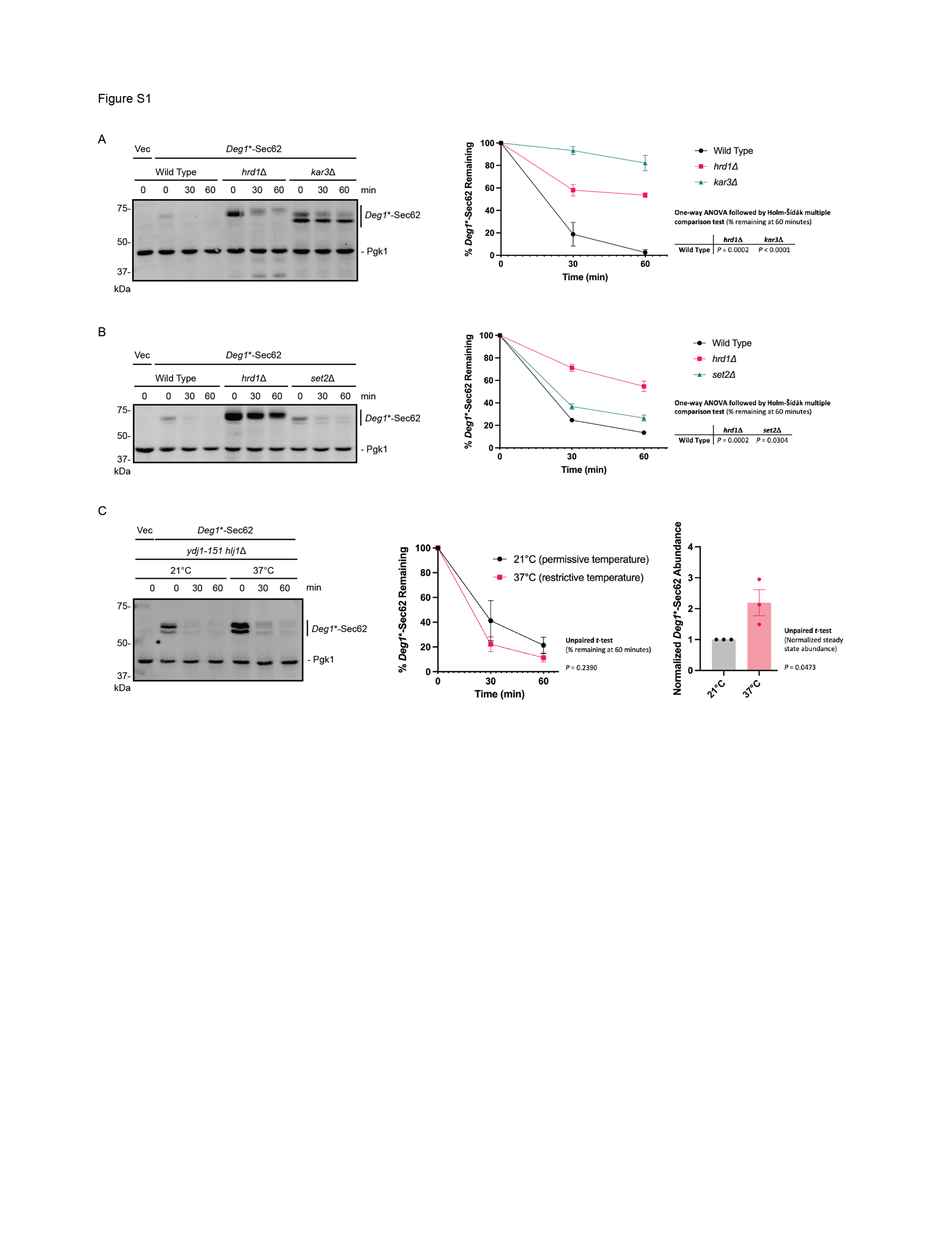
**

**Figure S1. Stabilization of *Deg1**-Sec62 in yeast lacking *KAR3* and *SET2*.** **(A and B)** Yeast of the indicated genotypes were transformed with a plasmid encoding *Deg1**-Sec62 or an empty vector and subjected to cycloheximide chase and western blot analysis to detect *Deg1**-Sec62 and Pgk1. Means of percent *Deg1**-Sec62 remaining at 60 minutes were evaluated by one-way ANOVA followed by Holm-Šídák multiple comparison tests (only pairs relative to wild type yeast were compared). **(C)** *ydj1-151* *hlj1*Δ yeast were transformed with an empty vector or a plasmid encoding *Deg1**-Sec62 and cultured at the permissive (21°C) or non-permissive (37°C) temperature for temperature-sensitive ydj1-1 function and subjected to cycloheximide chase and western blot analysis. Ydj1 and Hlj1 are partially redundant Hsp40 cochaperones (Caplan *et al.* 1992; Hrizo *et al.* 2007). Means of percent *Deg1**-Sec62 remaining (middle) and of normalized steady state (0 min) *Deg1**-Sec62 abundance (right) for 3 biological replicates are plotted. Means of percent remaining at 60 minutes and of normalized steady state *Deg1**-Sec62 abundance were plotted and evaluated by unpaired, two-tailed *t*-tests. **(A-C)** Error bars represent Standard Error of the Mean.

**
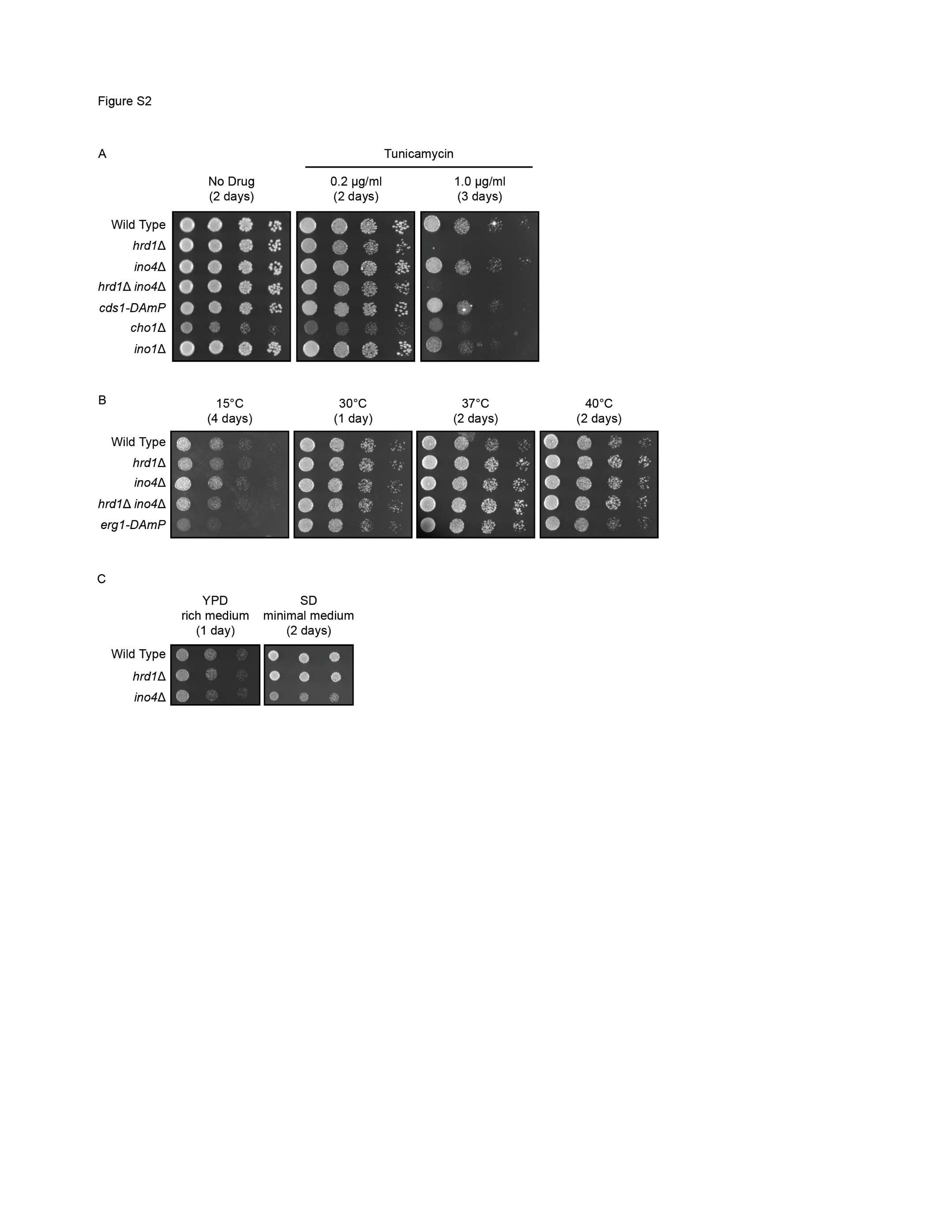
**

**Figure S2. Phenotypic analysis of yeast defective for lipid biosynthesis.** Yeast of the indicated genotypes were serially diluted and spotted onto agar plates. Plates were incubated at 30°C or the indicated temperatures and imaged after 1-4 d. Plates in **(A)** and **(B)** contain rich (YPD) yeast medium. Plates were incubated at 30°C **(A, C)** or the indicated temperatures **(B)** or and imaged after 1-4 d. Experiments were performed in triplicate (i.e. 3 biological replicates).

**
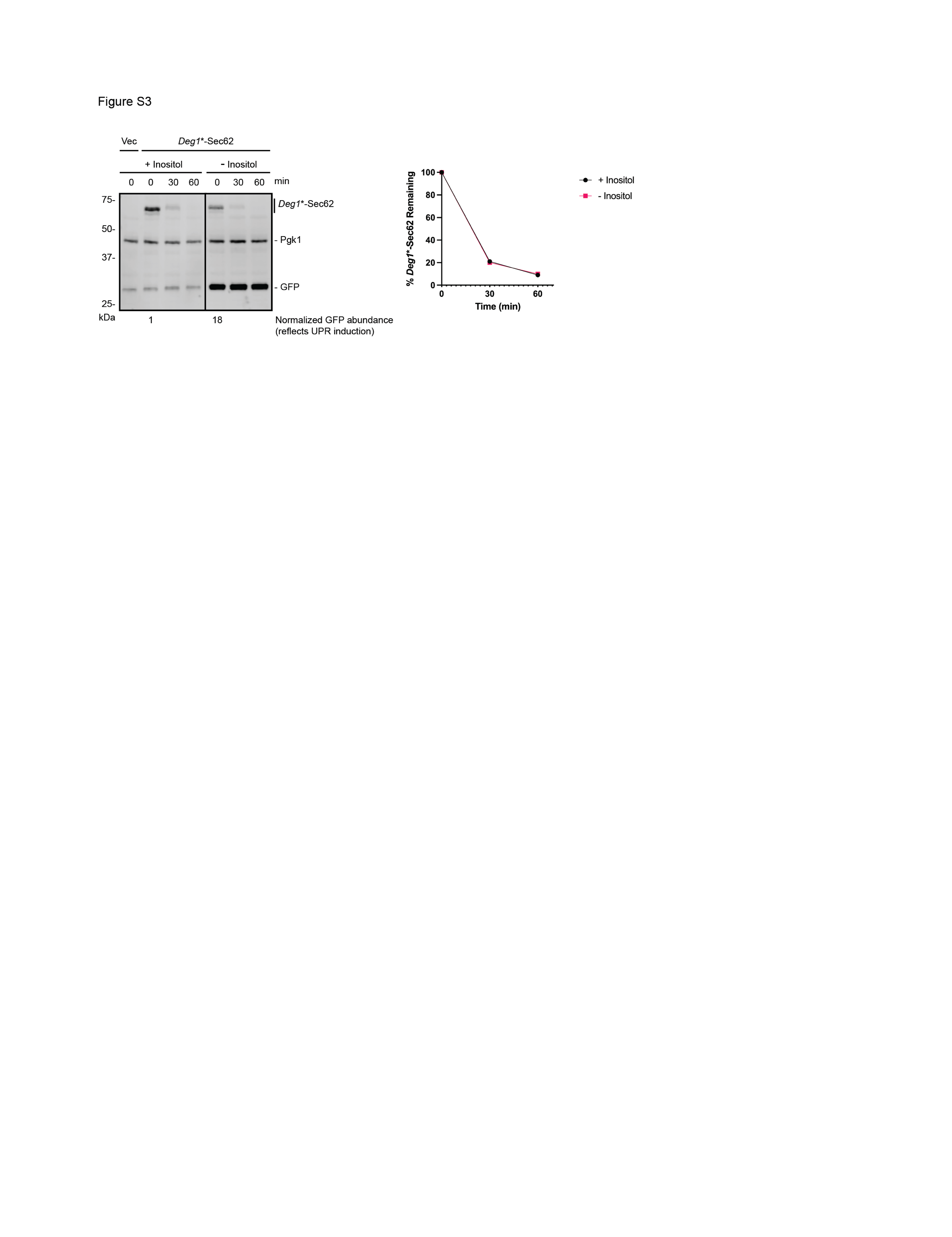
**

**Figure S3. Short-term inositol limitation does not impair *Deg1**-Sec62 degradation.** Wild type yeast were transformed with a plasmid encoding *Deg1**-Sec62 or an empty vector as well as a plasmid encoding GFP driven by the unfolded protein response element (UPRE). Cells were cultured to mid-exponential phase, washed six times in medium with inositol (+ inositol) or lacking inositol (- inositol), resuspended in the same media, cultured for 5 h at 30°C, and subjected to cycloheximide chase and western blot analysis to detect *Deg1**-Sec62, GFP, and Pgk1. Increase in GFP abundance following inositol limitation reflects induction of the unfolded protein response (UPR) (Promlek *et al.* 2011). Right, quantification of experiment. This experiment, which replicates results in (Buchanan *et al.* 2019), was performed one time.

**SUPPORTING FILE**

**File S1. Results of genome-wide screen.**
